# Multidimensional Effectiveness of Botulinum Toxin in Neuropathic Pain: A Systematic Review of Randomized Clinical Trials

**DOI:** 10.3390/toxins14050308

**Published:** 2022-04-27

**Authors:** Lorenzo Lippi, Alessandro de Sire, Arianna Folli, Francesco D’Abrosca, Elisa Grana, Alessio Baricich, Stefano Carda, Marco Invernizzi

**Affiliations:** 1 Physical and Rehabilitative Medicine, Department of Health Sciences, University of Eastern Piedmont “A. Avogadro”, 28100 Novara, Italy; lorenzolippi.mt@gmail.com (L.L.); arianna.folli23@gmail.com (A.F.); fradabro@gmail.com (F.D.); alessio.baricich@med.uniupo.it (A.B.); 2 Translational Medicine, Dipartimento Attività Integrate Ricerca e Innovazione (DAIRI), Azienda Ospedaliera SS. Antonio e Biagio e Cesare Arrigo, 15121 Alessandria, Italy; 3 Physical Medicine and Rehabilitation Unit, Department of Medical and Surgical Sciences, University of Catanzaro Magna Graecia, 88100 Catanzaro, Italy; alessandro.desire@unicz.it; 4 Neuropsychology and Neurorehabilitation Service, Department of Clinical Neuroscience, Lausanne University Hospital, 1004 Lausanne, Switzerland; granaelisa@gmail.com (E.G.); stefano.carda@gmail.com (S.C.); 5 Physical and Rehabilitation Medicine, “Ospedale Maggiore della Carità” University Hospital, 28100 Novara, Italy

**Keywords:** botulinum toxin (BoNT), neuropathic pain, pain management, quality of life, rehabilitation

## Abstract

Although botulinum toxin (BoNT) has been suggested as a treatment to counter neuropathic pain, no previous systematic reviews investigated the multidimensional effects of BoNT on pain relief and Health-Related Quality of Life (HR-QoL). The aim of this systematic review is to summarize the current evidence on the effectiveness of BoNT treatment for neuropathic pain, and to characterize its multidimensional effectiveness in order to guide physicians in clinical practice. Five databases were systematically searched up to 4 April 2022, to identify randomized controlled trials satisfying the following criteria: adults suffering from neuropathic pain, BoNT administration, any comparator, multidimensional assessment of pain as primary outcome, HR-QoL, physical function, anxiety and depression, and sleep quality as secondary outcomes. Twelve studies were included. The multidimensional pain scales used were short-form McGill Pain Questionnaire, Neuropathic pain scale, Neuropathic Pain Symptom Inventory, International SCI Pain Basic Data Set, West Haven-Yale Multidimensional Pain Inventory, Brief Pain Inventory, and Douleur Neuropathique 4. These scales highlighted the positive effects of BoNT administration. According to the Jadad scale, all the RCTs included were high-quality studies. BoNT administration might be effectively introduced in the comprehensive management of neuropathic pain. Further research should focus on optimal and cost-effective therapeutic protocols.

## 1. Introduction

Pain is currently defined by the International Association for the Study of Pain (IASP) as ‘an unpleasant sensory and emotional experience associated with, or resembling that associated with, actual or potential tissue damage’ [1]. Among the different pain types, neuropathic pain is characterized by increased pain sensitivity and/or spontaneous pain and is defined by the presence of neuropathy, a lesion or disease affecting the somatosensory nervous system [1]. It is currently considered a challenge in the clinical setting due to its chronic course and poor responsiveness to medications [2,3,4,5]. In further detail, the recent systematic review by van Hecke et al. [6] reported that approximately 6.9–10% of the European population suffer from neuropathic pain, with detrimental consequences in terms of physical and psychosocial wellbeing, health-related quality of life (HR-QoL) and economic burden [3,7,8].

Pathophysiology of neuropathic pain has been widely investigated and the present evidence underlines the role of independent mechanisms triggered by various damages to an afferent pathway [5,9,10,11,12]. However, the exact pathophysiological mechanisms underpinning neuropathic pain are far from being fully understood [13]. In this context, the downregulation of sodium channels [14], the dysregulation of Transient Receptor Potential Vanilloid 1 (TRPV1) and Transient Receptor Potential Member 8 (TRPM8) receptors have been proposed to have a role in this complex framework [15,16,17]. Interestingly, it has recently been highlighted that abnormal sensory messages characterizing neuropathic pain might stimulate the cortex, promoting the excitation of neurons in the limbic areas related to anxiety, depression, and sleep problems, frequently accompanying neuropathic pain [5]. A deeper assessment of these pathological mechanisms might play a key role in optimizing a multidimensional treatment, selecting a precise pathophysiological pathway [3,18]. In this complex framework, multimodal therapeutic interventions targeting specific structures involved in the neuropathic pain circuits are crucial to promoting the optimal response in pain relief [5]. Furthermore, several pharmacological and non-pharmacological approaches have been proposed for the complex management of neuropathic pain and growing evidence recommends a comprehensive patient-centered approach in order to improve pain management and minimize the side effects of single therapies [3,19,20,21,22].

In the last decade, botulinum toxin (BoNT) has been proposed as a therapeutic option to treat neuropathic pain [23], although the antinociceptive effects of BoNT have been widely ascribed to the muscle relaxation effects alone [24,25]. However, several studies reported positive results of BoNT treatment in the management of neuropathic pain [26,27,28]. Park et al. [28], in a neuropathic pain animal model, demonstrated the dissociation between the duration of muscle relaxation and pain relief after BoNT treatment, suggesting a pure antinociceptive role. 

Despite these findings, the recent GRADE classification by Finnerup et al. [29] reported BoNT as a third-line pharmacological treatment in general neuropathic pain. They suggested gabapentin, pregabalin, SNRIs (duloxetine/venlafaxine), and tricyclic antidepressants as first-line treatments, followed by capsaicin patches, lidocaine patches and tramadol. Similarly, the guidelines for neuropathic pain published by Moisset et al. [30] in 2020 recommended the use of BoNT as second-line therapy for peripheral neuropathic pain, while lidocaine plasters or Transcutaneous Electrical Nerve Stimulation (TENS) therapy were the first-line interventions. On the other hand, SNRI drugs, gabapentin, or tricyclic antidepressant were recommended for the treatment of central neuropathic pain, while pregabalin, tramadol, or combination therapy were recommended as second-line therapy. 

Although BoNT has been suggested as an effective treatment to counter neuropathic pain [29,30,31,32,33,34,35], evidence in the literature is mainly focused on the unidimensional evaluation of pain, with different systematic reviews assessing the Visual Analogue Scale (VAS) or Numerical Rating Scale (NRS). Conversely, given the psychosocial and functional burden of neuropathic pain, a multidimensional assessment of this condition is needed, in order to promote a patient-centered approach.

However, to the best of our knowledge, no previous systematic reviews investigated the multidimensional effectiveness of BoNT on pain relief and quality of life in patients suffering from neuropathic pain.

Therefore, this systematic review of randomized controlled trials (RCTs) aimed at summarizing the current evidence on the efficacy of BoNT treatment for neuropathic pain, characterizing the multidimensional effectiveness of BoNT related to different neuropathic pain etiologies to improve the complex management of this burdensome condition.

## 2. Results

**Figure 1:**
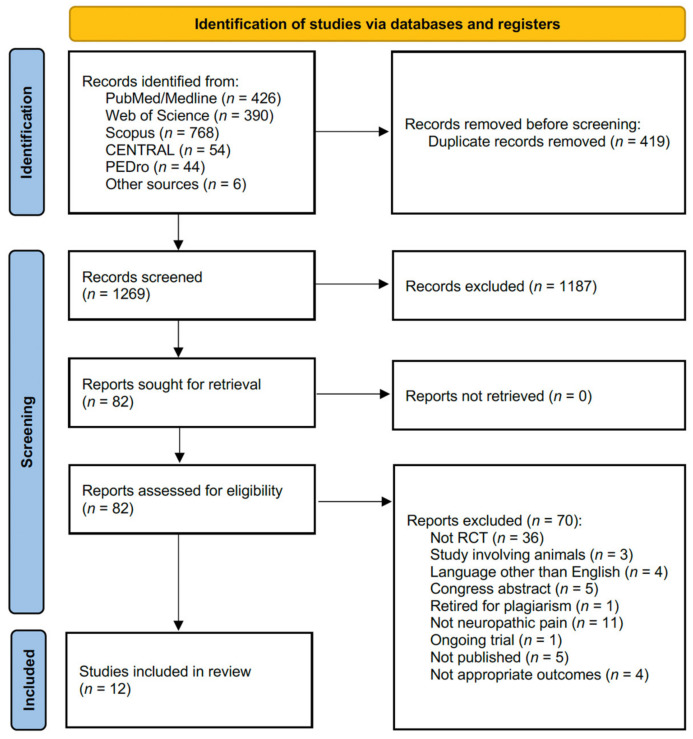
PRISMA 2020 flow chart.

The search strategy performed on 4 April 2022 identified 1688 records from the five databases and six records from the reference lists of the included studies. Figure 1 shows the PRISMA flow diagram of the search process. After duplication removal, 1269 studies were assessed for eligibility and screened for title and abstract. After the exclusion of 1187 records, 82 full-text records were assessed for eligibility. Due to inconsistency with the eligibility criteria, 70 articles were excluded (36 were not RCTs, 3 studies involved animals, 4 were in a language other than English, 1 was retired for plagiarism, 11 did not assess patients with neuropathic pain, 1 was an ongoing trial, 5 were congress abstracts, 5 were registered protocols not published, and 4 did not assess appropriate outcomes). The Supplementary Table S2 shows the lists of excluded studies assessed in full text and the reasons for exclusions. As a result, 12 studies were included in the present work [26,36,37,38,39,40,41,42,43,44,45,46]. The studies included in this systematic review were published between 2006 [46] and 2020 [43]. Among these, two studies were conducted in the USA [37,46]; three studies were conducted in Iran [39,42,43]; one study was conducted in Taiwan [45]; one was conducted in Greece [36]; one was conducted in Canada [38]; one was conducted in South Korea [40]; one was conducted in France [41]; and one was conducted in China [44]. The remaining study was an international collaboration (France and Brazil, n = 1 [26]). 

A total of 522 subjects were included in the present systematic review (90 with postherpetic neuropathic pain [36,44], 48 with spinal cord injuries [37,40], 29 with posttraumatic/postoperative nerve lesion or post-herpetic neuropathy [41], 66 with peripheral nerve lesion [26], 231 with diabetic neuropathy [39,42,43,45], 38 with thoracic outlet syndrome [38], and 20 with carpal tunnel syndrome [46]). The mean age of the subjects included ranged from 36.8 ± 8.9 years [38] to 77.5 ± 8.2 years [36], while 230 patients were males and 272 females. However, it should be noted that Breuer et al. [46] did not report the mean age and sex of the study participants. 

The intervention was compared to placebo or other treatments. In particular, the control group in each study was composed of patients suffering from neuropathic pain with the same etiological cause of the intervention group [26,36,37,38,39,40,41,42,43,44,45,46]. Control groups were treated with normal saline injections in 11 studies [26,36,37,38,39,40,41,42,43,45]. Only Xiao et al. [44] compared BoNT-A injection to both an active control group (treated with 0.5% lidocaine injection) and a placebo group (treated with saline injection). Two studies were crossover studies [37,45]. 

The time of follow-up varied somewhat among the studies included with five studies reporting a total duration of 24 weeks [26,36,38,41,45], one study of 20 weeks [37], two studies of 12 weeks [42,44], one study of 13 weeks [46], one study of 8 weeks [40], one study of 4 weeks [43], and one study of 3 weeks [39]. The sample characterization of each study included has been summarized in detail in Table 1. 

In conclusion, it should be noted that among the 12 RCTs included in the present review, 3 studies [36,42,43] did not report any funding, while 9 studies [26,37,38,39,40,41,44,45,46] received external funding and, in particular, 3 studies [38,40,46] received funding by pharmaceutical companies. Lastly, 2 studies [37,41] declared that they were supplied with the toxin by pharmaceutical companies.

### 2.1. BoNT Intervention

A high heterogeneity of BoNT type, source, amount, injection sites, number of injections and injection technique was reported in the studies included in the present work. Out of the 12 RCTs assessed, 11 (91.7%) [26,36,37,38,39,40,41,42,43,44,45] utilized BoNT-A, while 1 (8.3%) [46] administered BoNT-B.

Two studies assessed patients with postherpetic neuropathy with an amount of BoNT-A in the study protocols ranging between 100 and 200 units [36,44]. Both study protocols assessed the effects of BoNT-A injected subcutaneously in the painful area. In further detail, one of the two administration protocols was characterized by a chessboard distribution of 40 different injections, with a minimum distance of 1 cm between injection sites [36]. The other study assessed injections every 1.0–2.0 cm radius of skin [44].

Only one study protocol has been proposed in patients with peripheral nerve lesions and was characterized by two subcutaneous injections of up to 300 units of OnabotulinumtoxinA performed after 12 weeks [26]. The maximum number of injections was 60, all performed at a distance of 1.5–2 cm [26].

Similarly, the study by Ranoux et al., assessed the effects of subcutaneous injections (maximum 200 units) of OnabotulinumtoxinA in patients with post-traumatic/postoperative nerve lesion or postherpetic neuropathy [41]. The authors performed up to 40 different subcutaneous injections at a distance of 1.5 cm [41].

One study assessed the effects of 75 units of OnabotulinumtoxinA in the anterior and middle scalene muscles in patients with neuropathic pain due to thoracic outlet syndrome [38]. The intramuscular injections were performed under EMG guidance [38].

The effects of BoNT-A in patients with diabetic neuropathy have been studied by four different RCTs with different injection protocols. All the studies included administered BoNT-A subcutaneously. One study performed an injection of 50 units of OnabotulinumtoxinA [45] while two studies injected 100 units of AbobotulinumtoxinA [39,42]. BoNT-A was administered in the sole, dorsum or entire surface of the foot. The injections number ranged between 12–20 *sites* per patient [39,42,43,45].

In patients with neuropathic pain due to Spinal Cord Injury (SCI), two studies assessed different protocols of subcutaneous administration of BoNT-A in the painful area. The amount of BoNT-A ranged between 200 and 400 units [37,40]. One study performed 5 units per injection site [37] and the other did not report the single injection amount of BoNT-A [40].

Lastly, one study injected BoNT-B in patients with carpal tunnel syndrome [46], dividing 2500 units of RimabotulinumtoxinB into three intramuscular injections (one for each muscle), under EMG guidance to identify opponens digiti minimi and flexor digiti minimi, and anatomically locating palmaris brevis muscle [46].

All the BoNT administration protocols are summarized in detail in Table 2.

### 2.2. Main Findings

#### 2.2.1. Primary Outcome-Multidimensional Pain Assessment

The primary outcome assessed in this review was the effectiveness of BoNT injections in terms of multidimensional pain scales and were assessed in eight studies [26,37,39,40,41,42,43,46].

In further detail, three studies [39,42,43] assessed pain with the Neuropathic Pain Scale (NPS), showing significant improvements in most of the subitems considered [39,42,43]. The Neuropathic Pain Symptom Inventory (NPSI) has been used in two RCTs, reporting significant improvements in specific subitem scales (burning [26,41], paroxysmal [26], electric shock [41] and evoked pain to cold [41]). However, Ranoux et al. [41] did not show significant NPSI modifications 24 weeks after BoNT-A treatment. Similarly, these studies [26,41] assessed the Brief Pain Inventory (BPI) scale, reporting significant differences (*p* < 0.05) compared to placebo, but only in terms of pain intensity subitems [26,41].

Interestingly, Chun et al. [37] assessed pain intensity with the International SCI Pain Basic Data Set. The authors reported that 33% of patients assessed showed a significant change in pain intensity at 8 and 12 weeks, 50% showed a decreased pain interference with daily activities at 2 and 4 weeks, 50% reported a reduced pain interference with mood at 2 weeks, 33% at 4 and 8 weeks, and 50% reported a reduced pain interference with sleep at 2 and 4 weeks, 17% at 8 and 12 weeks. On the other hand, it should be noted that the statistical analysis was based on a descriptive approach [37].

In contrast, Han et al. [40] investigated the effectiveness of BoNT-A administration with the short-form McGill Pain Questionnaire, reporting significant differences between groups at 4 weeks (*p* < 0.05) and 8 weeks (*p* < 0.05) [40]. The Douleur Neuropathique 4 question scale has been used to assess pain in patients with diabetic neuropathy, showing significant improvement in electric shocks (*p* = 0.01), burning (*p* < 0.01), pins and needles (*p* = 0.03) and brushing (*p* < 0.001) subitems [39].

Lastly, Breuer et al. [46] assessed pain intensity with the West Haven-Yale Multidimensional Pain Inventory, highlighting improvements in quality-of-life indicators, reaching significance in some of the different time points assessed (*p* < 0.05). However, no significant differences between groups were reported (*p* = NS) [46].

#### 2.2.2. Secondary Outcomes

The secondary outcomes assessed in the present systematic review were HR-QoL, physical function, anxiety and depression, and sleep quality.

In particular, HR-QoL has been assessed in six studies [26,37,38,40,42,45]. SF-36 has been assessed in three RCTs [38,42,45] reporting controversial results. In further detail, in patients with diabetic neuropathy, the RCT by Salehi et al. [42] reported significant improvement of SF-36 (*p* = 0.007) [42], while Yuan et al. [45] did not demonstrate significant differences between groups (*p* = NS) [45]. On the other hand, the study by Finlayson et al. [38] did not report significant improvement in SF-36 (*p* = NS) after BoNT-A treatment [38]. The World Health Organization Quality of Life questionnaire (WHOQOL-BREF) has been proposed by Han et al. [40] to assess pain relief in patients with SCI undergoing BoNT-A treatment; however, no significant differences were reported in the four domains of WHOQOL-BREF after the BoNT-A intervention [40]. On the other hand, Attal et al. [26] assessed EQ5D VAS scale, without underlining significant differences between groups (*p* = NS). Lastly, Chun et al. [37] reported at least moderate improvements in QoL in 33% of patients assessed at 2, 4, and 12 weeks, 17% at 8 weeks. Unfortunately, descriptive statistics was performed, without assessing the significance of the reported results [37]. Interestingly, physical function was assessed by Finlayson et al. [38] by the Disabilities of the Arm, Shoulder, and Hand scale; however, the authors did not report significant improvement (*p* = NS) after BoNT-A treatment [38].

On the other hand, anxiety and depression have been assessed through the Hospital Anxiety and Depression Scale (HADS) in two studies [26,41]: there were no significant differences between the BoNT-A and the placebo group (*p* = NS) in both studies [26,41].

Sleep quality has been specifically studied in six RCTs [26,36,42,44,45,46] assessing patients with herpetic neuropathy [36,44], peripheral nerve lesions [26], diabetic neuropathy [42,45] and carpal tunnel syndrome [46]. Thus, a high heterogeneity of the outcome measures was reported. The Pittsburgh Sleep Quality Index (PSQI) has been used in two studies [42,45] performed on patients with diabetic neuropathy. In further detail, the study by Salehi et al. [42] reported significant differences in the PSQI after the BoNT-A intervention compared to the placebo group [42]. On the contrary, in the RCT by Yuan et al. [45], the difference in the improvement in sleep quality between the BoNT-A group and the placebo group reached significance (1.72 ± 1.82 vs. −0.11 ± 2.78, *p* < 0.05) exclusively at 4 weeks after intervention. [45]. The Sleep Problem Index has been used by Attal et al. [26], reporting significant differences (Sleep Problem Index I, six items: 43.9 ± 21.4 vs. 40.6 ± 20.6; *p* = 0.02; Sleep Problem Index I, nine items: 45.3 ± 19.3 vs. 41.7 ± 19.6; *p* = 0.03) in the intergroup analysis [26]. Similarly, the study by Apalla et al. [36] assessed sleep quality with a five-item questionnaire in 14 patients with post-herpetic neuropathy treated with BoNT-A, showing significant improvements at week 2 (*p* < 0.001) and week 4 (*p* < 0.001), compared to placebo [36]. Sleep time has been also assessed by Xiao et al. [44], showing a significant improvement at day 7 and after 3 months from the BoNT-A treatment (*p* < 0.01) in patients with postherpetic neuropathy compared with lidocaine and placebo groups [44].

Lastly, the RCT by Breuer et al. [46] assessed sleep interference by pain in patients with carpal tunnel syndrome, reporting significant improvements (*p* < 0.05) in some of the time points assessed. On the other hand, the authors did not find a statistically significant difference between groups (*p* = NS) [46].

Table 1 reported further detail of the main results of the RTCs included in the present review.

### 2.3. Study Quality

According to the Jadad scale [47], all the RCTs included (n = 12, 100%) were high quality studies [26,36,37,38,39,40,41,42,43,44,45,46]. Table 3 showed in detail the score of each subitem of the Jadad scale for the RCTs included.

**Figure 2:**
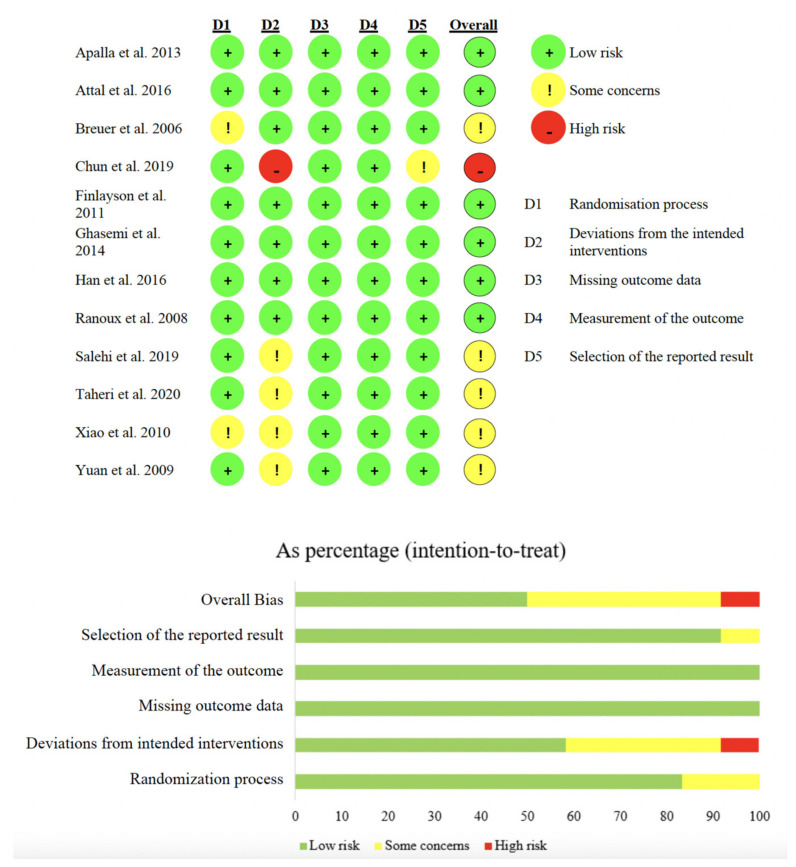
Risk of bias summary of the included studies.

The risk of bias assessed by RoBv.2 [48] showed that 10 studies (83.3%) [26,36,37,38,39,40,41,42,43,45] ensured correct randomization, while 4 studies (33.3%) [42,43,44,45] showed some concerns in the second domain due to the lack of details about the blinding of the study participants. One study (6.7%) resulted in high risk of bias because it did not reach the target sample size [37]. All studies (n = 12, 100%) [26,36,37,38,39,40,41,42,43,44,45,46] showed low risk of bias in missing outcome data and outcome assessment, and 11 studies (91.7%) showed low risk of bias in selection of the reported results. See Figure 2 for further details.

## 3. Discussion

To date, there is a lack of consensus about the multidimensional effectiveness of BoNT in neuropathic pain and the optimal BoNT administration protocols are still debated [49,50,51,52].

Our findings showed a significant effect of BoNT administration in patients suffering from neuropathic pain due to postherpetic neuralgia [36,44], SCI [37,40], peripheral nerve lesion [26], diabetic neuropathy [39,42,43,45], post-traumatic/postoperative neuropathies [41], and carpal tunnel syndrome [46]. Our study results are in accordance with other evidence reporting positive effects of BoNT on pain management of non-specific neuropathic pain [51,52,53]. On the contrary, the etiological cause of neuropathic pain seems strictly related to the treatment effectiveness. In particular, Finlayson et al. [38] did not show a statistically significant improvement in patients with thoracic outlet syndrome pain [38]. Accordingly, Breuer et al. [46] did not reveal any significant differences between BoNT-B administration and placebo group in carpal tunnel syndrome, suggesting that BoNT might not provide additional benefits in the management of neuropathic pain with nerve compression etiology [38,46]. In contrast, most of the studies included highlighted positive results in the multidimensional management of neuropathic pain in several pathological conditions [26,36,37,39,40,41,42,43,44,45]. However, it should be noted that all these studies [26,36,37,39,40,41,42,43,44,45] assessed the effectiveness of BoNT-A administration, while the RCT by Breuer et al. [46] was the only study that assessed BoNT-B; therefore, the role of BoNT-B in neuropathic pain management is far from being fully characterized.

As a result, clinicians should be aware of the evidence supporting BoNT use in specific conditions and the therapeutic intervention should be based on a precise diagnosis in order to select the patients more suitable to achieve better pain relief. Interestingly, our data showed positive long terms results of BoNT compared to lidocaine injections [44] in patients with post-herpetic neuropathy. These controversial data might be related to the characteristics of neuropathic pain and the BoNT administration protocols that were often heterogeneous in the studies included in the present review [26,36,37,38,39,40,41,42,43,44,45,46].

In this scenario, the current literature underlines a large gap of knowledge regarding the optimal BoNT therapeutic strategy, and this might be related to the lack of standardized BoNT administration protocols and injective techniques [54,55,56]. On the other hand, the French Recommendation for Neuropathic Pain of 2020 [30] provided a general indication of dosage from 50 to 300 units (onabotulinumtoxinA) every 3 months, without fully characterizing the intervention protocols or without suggesting any differences based on patients’ characteristics. Our findings showed that although the maximum amount of BoNT-A injection might reach 400 units [37] and BoNT-B might reach 2500 units [46], specific subgroup analysis based on neuropathic pain should be considered and a wide difference in the dosage injected based on patient characteristics has been reported [26,36,37,38,39,40,41,42,43,44,45]. Similarly, in the past few years, different narrative and systematic reviews assessed the effects of BoNT administration characterizing patients with different types of neuropathic pain [49,51,52].

In particular, the systematic review by Hary et al. [57] assessed the effectiveness of BoNT-A administration in terms of pain relief in patients with peripheral neuropathic pain [57]. The authors reported significant effects in VAS scores, reporting better results in patients with diabetic polyneuropathy compared to patients with postherpetic, posttraumatic, or postsurgical neuralgia at 1 and 3 months post injection [57]. However, the authors mainly focused on unidimensional pain assessment and sleep improvement [57].

On the other hand, it should be noted that unidimensional scales lack the ability to characterize pain as a complex personal experience: these measurements heavily weight not only patient treatment satisfaction but also physician decision making [58]. In this context, multidimensional pain scales might better characterize pain intensity, nature, and location, and its consequences in function or mood, producing a quantitative description aiming at becoming the preferential assessment in a holistic approach [58]. Therefore, to the best of our knowledge, the present work represents the first systematic review of RCTs summarizing the current evidence on specific BoNT administration protocols providing data about the multidimensional effectiveness based on etiological cause of neuropathic pain to guide physicians in effective and safe therapeutic interventions in clinical practice.

Moreover, the large heterogeneity of administration protocols raises questions about the need to identify the lowest effective dose, not only to minimize the risk of adverse events but also from a cost effectiveness standpoint [59,60,61]. In particular, given the high prevalence of neuropathic pain and the strictly related sanitary costs [6,62], cost-effective therapies are mandatory in large-scale interventions aiming at improving quality of life and well-being of these patients. None of the studies included in this systematic review provided a precise cost analysis to better address the critical issue of the sanitary costs of pain management [26,36,37,38,39,40,41,42,43,44,45,46]. Therefore, future research is needed to better address this emerging issue in the clinical scenario.

Although unidimensional assessment has been proposed in the current literature to provide relevant quantitative data about BoNT efficacy [3,8,63], to date, clear evidence in multidimensional assessment for neuropathic pain is still lacking. In the present work, we sought to assess the effects of BoNT-induced pain relief in HR-QoL of patients with neuropathic pain. Unfortunately, our results revealed conflicting evidence regarding supporting BoNT efficacy on the overall well-being of patients suffering from neuropathic pain [26,36,37,38,39,40,41,42,43,44,45,46]. Nevertheless, it should be noted that recent research is now focused on a multidisciplinary framework with growing evidence emphasizing the need for comprehensive and synergic treatments to improve outcomes of patients with chronic neuropathic pain [3,20,64,65].

In this complex scenario, several therapeutic approaches have been investigated and might be proposed to improve pain management in patients with chronic neuropathic pain, including mini-invasive interventions [22,66,67,68], pharmacological drugs [67,69,70], nutraceuticals [71,72], physical exercise [21,73,74] and instrumental rehabilitative techniques [20,75]. In addition, recent advances in understanding the pathophysiological mechanisms underpinning pain chronification reveals that specific peripheral [11,12,76] and central circuits might be involved [12,77]. In further detail, it has been reported that after peripheral nerve damage, sodium channels increase in quantity in both the involved fibers and surrounding ones, which might lower the action potential threshold of the stimulus [14]. Hence, pain in the absence of an external stimulus might be due to an ectopic signal generated along this pathway [78,79,80]. In the hyperalgesia state, some receptors like TRPV1, involved in the noxious heat pathway [15], and the receptor TRPM8 involved in the cold pathway are upregulated [16,17]. Furthermore, it has been proposed that central sensitization mechanisms might affect continuous discharge of peripheral afferent fibers in the dorsal horn of the spinal cord inducing structural modifications in postsynaptic neurons [9,11]. Other contributors to pain hypersensitivity after a nerve lesion are inflammation, loss of inhibitory GABAergic interneurons in the spinal horn and enhanced sympathetic activity [9]. Even if the precise mechanism of action of BoNT is far from being understood in detail [77], recent research suggested a possible role in the nociceptive peripheral pathway, inflammation and even in central activities related to retrograde axonal transport to the spinal cord [81]. BoNT might implement HR-QoL in patients affected by neuropathic pain and might be considered as a part of a comprehensive management strategy including both pharmacological and non-pharmacological approaches [82,83]. However, to date, the effects of combined interventions to treat neuropathic pain have not been studied in the current literature; indeed, the role of BoNT injections in integrated multitarget interventions still remains unknown.

Taken together, the findings of this systematic review of RCTs might improve the knowledge about the possible role of BoNT treatment in chronic neuropathic pain [84]. In addition, the data reported by the RCTs included in the present review might support previous evidence suggesting positive effects of BoNT in patients with neuropathic pain [54,55,56], underlining the effectiveness of specific administration protocols tailored to patient characteristics.

Despite these considerations, we are aware that the present systematic review is not free from limitations. In particular, the lack of meta-analysis represents the main limitation of the present work. Unfortunately, the large heterogeneity of participants, intervention and outcomes assessed did not allow performance of a quantitative analysis, in accordance with the Cochrane Handbook for Systematic Review of Intervention (Ver, 6.1, 2020) [85]. Moreover, our search strategy might not include all records in these field and other sources have been searched in accordance with the PRISMA 2020 guidelines [86] in order to cover the relevant literature related to this topic. Lastly, 5 out of 12 studies included were directly supplied by pharmaceutical companies distributing the BoNT. Therefore, potential conflicts of interests in the studies included should be considered before making strong conclusions.

## 4. Conclusions

To date, the mechanisms underpinning the therapeutic role of BoNT in neuropathic pain are not completely understood, but the RCTs included in the present systematic review showed promising results in terms of pain relief, suggesting that BoNT-A might effectively improve symptoms in patients with neuropathic pain.

Although our findings provided evidence about the current BoNT protocols for specific neuropathic pain treatment, this systematic review of RCTs underlined the need for high-quality studies to better elucidate the optimal and cost-effective therapeutic strategies of BoNT administration.

Therefore, further evidence with standardized BoNT protocols in deeply characterized populations is needed to provide strong conclusions aiming to guide clinicians to implement precise and tailored treatment to improve the management of neuropathic pain.

## 5. Materials and Methods

### 5.1. Registration

This systematic review of randomized controlled trials (RCTs) was performed ethically in accordance with the Preferred Reporting Items for Systematic Reviews and Meta-analyses (PRISMA) statement [86]. The study protocol was realized before study initiation and submitted to PROSPERO (https://www.crd.york.ac.uk/prospero; accessed on 4 April 2022) with registration number CRD42022299703.

### 5.2. Search Strategy

We systematically searched PubMed/Medline, Scopus, Cochrane Central Register of Controlled Trials (CENTRAL), Physiotherapy Evidence Database (PEDro), and Web of Science for RCTs published up to 9 December 2021. Two investigators independently searched the databases. The search strategy is reported in Supplementary Table S1.

### 5.3. Selection Criteria

In accordance with the PICO model [87], we considered eligible RCTs satisfying the following criteria:

(P) Participants: adults suffering from neuropathic pain.
(I) Intervention: BoNT type A (BoNT-A) or BoNT type B (BoNT-B) administration.
(C) Comparator: any comparator, including placebo, other pharmacological treatment, non-pharmacological treatment or no treatment.
(O) Outcome: the primary outcome was self-reported pain relief in terms of multidimensional pain scales. The secondary outcomes were HR-QoL, physical function, anxiety and depression, and sleep quality.

We included RCTs published in peer-reviewed international journals in the English language. The exclusion criteria were the following: (i) studies involving animals; (ii) language other than English; (iii) participants with pregnancy; (iv) conference abstracts.

After duplication removal, two investigators independently reviewed the title and abstracts of retrieved articles to choose relevant articles. Any discordance was resolved by collegial discussion. If consensus was not achieved, a third reviewer was asked.

### 5.4. Data Extraction and Synthesis

All data were assessed and extracted by two authors independently from full-text documents into Microsoft Excel (IBM, New York, NY, USA). Missing data was directly requested from corresponding authors. Any disagreement between the two reviewers was solved by collegial discussion among the authors. In case of disagreement, a third author was asked.

The following data were extracted: (1) title; (2) authors; (3) publication year; (4) nationality; (5) participants (number, mean age and age range, gender); (6) interventions’ characteristics; (7) comparator; (8) outcomes; (9) main findings; (10) funding.

The data extracted were summarized in tables. Subgroup analysis was performed based on neuropathic pain characteristics and by the BoNT administration modalities.

### 5.5. Quality Assessment and Risk of Bias

The quality of the studies included was assessed independently by two of the authors, according to the Jadad scale [47]. Discordances were solved by discussion between the authors or by asking a third reviewer. A Jadad score between 3 to 5 points was considered high quality.

The risk of bias was assessed by the Cochrane risk-of-bias tool for randomized trials (RoBv.2) [48]. Bias was classified as low, high, or unclear based on the item of RoBv.2. In particular, the domains assessed by RoBv.2 were: (i) random process; (ii) deviation from the intended interventions; (iii) missing outcome data; (iv) measurement of the outcome; and (v) selection of the reported result.

## Figures and Tables

**Table 1:** Characteristics of the RCTs included.

Article	Design	Intervention Characteristics	Comparison	Sample Size		Outcomes Measures	Follow-Up	Main Findings	Indications
				*Intervention*	*Comparator*				
Apalla et al. [36]	Randomized, double-blind, placebo-controlled clinical trial	Forty injections of Onabotulinumtoxin-A (100 units) in the painful area	Placebo (normal saline) injections	Patients with post-herpetic neuropathy *n*: 15 male/female: 8/7 Mean age: 73.2 ± 10.5	Patients with post-herpetic neuropathy *n*: 15 male/female: 10/5 Mean age: 77.5 ± 8.2	Quality of sleep, assessed by five-item questionnaire with a score ranging from 0 to 3	24 weeks	*Intervention Group* Sleep scores: significant improvements at week 2, which remained stable between weeks 2 and 4; after the initial decline, sleep scores remain unchanged until week 12 *Comparator Group* Sleep scores: no significant improvement at week 2, which remained unchanged between weeks 2 and 4 *Between groups* Sleep scores: significant differences at week 2	BoNT-A administration significantly improves quality of sleep at 2 weeks.
Xiao et al. [44]	Randomized, double-blind, placebo-controlled clinical trial	BoNT-A group: administrations (comprising several injections) of LanbotulinumtoxinA (up to 200 units).	Lidocaine (active control) group: administrations (comprising several injections) of 0.5% lidocaine. Placebo group: placebo (normal saline) injections.	BoNT-A group: patients with postherpetic neuropathy *n*: 20 male/female: 11/9 Mean age: 70 ± 15.4	Lidocaine group: patients with postherpetic neuropathy *n*: 20 male/female: 8/12 Mean age: 65 ± 14.2 Placebo group: patients with postherpetic neuropathy *n*:20 male/female: 9/11 Mean age: 67 ± 12.1	Sleep time (hours)	3 months	*Intervention Group* Sleep time: significant improvement on day 7 and after 3 months *Comparator Group* Sleep time: significant improvement on day 7 and after 3 months in both lidocaine group and placebo group *Between groups* Sleep time: improvement of IG was significantly greater compared with lidocaine and placebo groups	BoNT-A administration significantly improves sleep time.
Attal et al. [26]	Randomized, double-blind, placebo-controlled, parallel-group clinical trial	Two administrations of Onabotulinumtoxin A (up to 300 units), 12 weeks apart.	Two administrations of saline, 12 weeks apart.	Patients with peripheral nerve lesion *n:* 34 male/female: 17/17 Mean age: 51.6 ± 16.7	Patients with peripheral nerve lesion *n:* 32 male/female: 20/12 Mean age: 52.3 ± 15.8	BPI, NPSI, EQ5D VAS, HADS, Sleep Problem Index	24 weeks	*Intervention Group* BPI VAS: significant reduction NPSI burning pain and paroxysmal pain subitem: significant improvement HADS, EQ5D VAS, Sleep Problem Index: *p* = NS *Comparator Group* BPI, NPSI, HADS, EQ5D VAS, Sleep Problem Index: *p* = NS *Between groups* BPI VAS: significant differences NPSI subscales: significant differences in paroxysmal pain and allodynia HADS: significant differences in anxiety Sleep Problem Index—6 items: significant differences Sleep Problem Index—9 items: significant differences	BoNT-A administration significantly improves of BPI VAS, NPSI burning pain and paroxysmal pain subitem, HADS, and Sleep Problem Index
Ranoux et al. [41]	Randomized, double-blind, placebo-controlled, parallel-group clinical trial	Administrations of Onabotulinumtoxin A (up to 200 units).	Administrations of saline.	Patients with posttraumatic/ postoperative or postherpetic neuropathy *n:* 15 male/female: 6/9 Mean age: 53.8 ± 13.9	Patients with posttraumatic/ postoperative or postherpetic neuropathy *n:* 14 male/female: 4/10 mean age: 49.7 ± 15.9	NPSI, BPI, HADS	24 weeks	*Intervention Group* NPSI subitems (burning, paroxysmal pain, allodynia): significant improvement at 12 weeks General activity and mood: improvement without significance HADS anxiety: slight improvement without significance *Comparator Group* NPSI, BPI, HADS: *p* = NS *Between groups* NPSI subscales (burning, electric shock and evoked pain to cold): significant differences at 12 weeks, without significant differences at week 24 BPI pain intensity: significant differences HADS: NS at 24 weeks	BoNT-A administration significantly improves NPSI subscales (burning, electric shock, and evoked pain to cold), and BPI pain intensity
Finlayson et al. [38]	Randomized, double-blind, placebo-controlled clinical trial	Seventy-five units of OnabotulinumtoxinA injected in the anterior and middle scalene muscles under EMG guidance.	Saline injected in the anterior and middle scalene muscles under EMG guidance.	Patients with thoracic outlet syndrome *n*: 20 male/female: 3/17 Mean age: 36.8 ± 8.9	Patients with thoracic outlet syndrome *n*: 18 male/female: 4/14 Mean age: 38.7 ± 7.0	DASH, SF-36	6 months	*Intervention Group* DASH, SF-36: not significant *Comparator Group* DASH, SF-36: not significant *Between groups* DASH, SF-36: not significant differences	BoNT-A administration did not improve DASH and SF-36
Ghasemi et al. [39]	Randomized, double-blind, placebo-controlled clinical trial	100 units of AbobotulinumtoxinA in 0.9% saline were injected, each injection approximately 8–10 units	Placebo (normal saline) injections	Patients with diabetic neuropathy *n:* 20 male/female: 9/11 Mean age: 62.7 ± 9.9	Patients with diabetic neuropathy *n:* 20 male/female: 13/7 Mean age: 59.3 ± 9.6	NPS and DN4 questionnaire.	3 weeks	*Intervention Group* NPS subitems: significant differences, except for cold sensation DN4 questionnaire subitems: significant improvement (electric shocks, burning, pins and needle, and brushing) *Comparator Group* NPS subitems: NS DN4 questionnaire subitems: NS *Between groups* NR	BoNT-A administration significantly improves NPS (except for cold sensation) and DN4 questionnaire subitems (electric shocks, burning, pins and needles, and brushing subitems)
Salehi et al. [42]	Randomized double-blind, placebo-controlled clinical trial	Twelve injections of AbobotulinumtoxinA (8.33 units each point) in the dorsal foot surface	Placebo (normal saline) injections	Patients with diabetic neuropathy *n*:16 male/female: 6/10 Mean age: 58.3 ± 5.3	Patients with diabetic neuropathy *n*:16 male/female: 6/10 Mean age: 56.7 ± 7.5	NPS, SF-36, and PSQI questionnaires.	12 weeks	*Intervention Group* NPS, SF-36 subitems: significant improvement PSQI: significant decrease *Comparator Group* NR *Between groups* SF-36: significant differences PSQI: significant differences NPS subitems: significant differences, except for sharp sensation, sensory sensation, and deep sensation	BoNT-A administration significantly improve SF-36, PSQI and NPS subitems (except for sharp sensation, sensory sensation, and deep sensation)
Taheri et al. [43]	Randomized, double-blind, placebo-controlled clinical trial	Group 1: twenty injections of BoNT-A (for a total of 150 units) in the sole of the right foot (7.5 units each injection); in the other feet, same procedure with saline placebo. Group 2: twenty injections of BoNT-A (for a total of 75 units) in the sole of both feet (3.75 units each injection, for a total of 150 units).	Placebo group: both feet with placebo (normal saline) injections.	Group 1: Patients with diabetic neuropathic pain *n*: 47 male/female: 16/31 Mean age: 54.5 ± 7.6 Group 2: Patients with diabetic neuropathic pain *n*: 47 male/female: 20/27 Mean age: 56.9 ± 6.2	Group N: Patients with diabetic neuropathic pain *n*: 47 male/female: 19/28 Mean age: 54.3 ± 8.2	NPS	4 weeks	*Intervention Group* Group 1: NPS subitems: significant improvements, except for dull sensation and cold sensation Group 2: NPS subitems: significant improvements, except for dull sensation (*p* = 0.622) and cold sensation *Comparator Group* NPS subitems: significant improvements, except for dull sensation, cold sensation (*p* = 0.067), unpleasant sensation and surface pain. *Between groups* NPS subitems: pain intensity, sharp sensation, hot sensation, sensitive sensation, unpleasant sensation, deep pain, and surface pain improved significantly after IGs vs. CG. Hot sensation subitem showed a significant difference between Group 1 vs. Group 2. Dull and cold sensations improvement did not show a significant difference between Group 2 and N.	BoNT-A administration significantly improve NPS (pain intensity, sharp sensation, hot sensation, sensitive sensation, unpleasant sensation, deep pain, and surface pain subitems)
Yuan et al. [45]	Randomized, double-blind, placebo-controlled, crossover clinical trial	OnabotulinumtoxinA injection of 50 units into each foot (4 units per injection); then crossover after 12 weeks.	Saline injection into each foot; then crossover after 12 weeks.	Patients with diabetic neuropathy *n:* 9 male/female: 6/12 Mean age: 65.6 ± 9.2	Patients with diabetic neuropathy *n:* 9 male/female: 6/12 Mean age: 65.6 ± 9.2	CPSQI, and SF-36	24 weeks	*Intervention Group* CPSQI: NS at week 12 *Comparator Group* NR *Between groups* CPSQI: significant improvements at 4 weeks SF-36: NS	BoNT-A administration significantly improve CPSQI
Chun et al. [37]	Randomized, double-blind, placebo-controlled, crossover clinical trial	Injection of up to 400 units OnabotulinumtoxinA (phase 1). After 12 weeks of follow up, cross-over of participants was performed and subcutaneous injection of normal saline was administered (phase 2, P2)	Injection of normal saline (placebo) (phase 1). After 12 weeks of follow up, crossover of participants was performed and subcutaneous injection of up to 400 units Onabotulinumtoxin A were administered (phase 2, P2)	Patients with SCI *n:* 8 male/female: 6/2 Mean age: 45 (32–61)	Patients with SCI *n:* 8 male/female: 6/2 Mean age: 45 (32–61)	ISCIPBDS and QOL	20 weeks	*Intervention Group* ISCIPBDS subitems: change in pain intensity at 8 and 12 weeks in 33% of patients. QOL: 33% of patients reported at least moderate improvements at 2, 4, and 12 weeks, 17% at 8 weeks *Comparator Group* ISCIPBDS subitems: no patient reported a change in pain intensity at 8 and 12 weeks. 17% reported decreased pain interference with daily activities at 2 and 4 weeks QOL: no changes at 2, 4, 8 and 12 weeks *Between groups* NR	BoNT-A administration improve ISCIPBDS subitems
Han et al. [40]	Randomized, double-blind, placebo-controlled clinical trial	200 units Letibotulinumtoxin A in 4 mL saline solution 1-time injection in painful area	Placebo (normal saline) injections	Patients with SCI *n:* 20 male/female: 15/5 Mean age: 53.1 ± 9.1	Patients with SCI *n:* 20 male/female: 14/6 Mean age: 48.9 ± 14.2	SF-MPQ and WHOQOL-BREF.	8 weeks	*Intervention Group* NR *Comparator Group* NR *Between groups* SF-MPQ: significant differences at 4 weeks and 8 weeks WHOQOL-BREF: NS	BoNT-A administration significantly improves SF-MPQ
Breuer et al. [46]	Randomized, double-blind, placebo-controlled clinical pilot trial	2500 units of rimabotulinumtoxin B in 0.5 mL of normal saline divided in 3 intramuscular under EMG guidance for opponens digiti minimi and flexor digiti minimi, and anatomically located for palmaris brevis muscle	Placebo (normal saline) intramuscular under EMG guidance for opponens digiti minimi and flexor digiti minimi, and anatomically located for palmaris brevis muscle	Patients with carpal tunnel syndrome *n:* 11 male/female: NR Mean age: NR	Patients with carpal tunnel syndrome *n:* 9 male/female: NR Mean age: NR	WHYMPI, Quality of sleep	13 weeks	*Intervention Group* WHYMPI quality of life indicators: improvements with statistical or borderline significance at different time points Pain interference with sleep (assessed with diary report): improved for weeks 2 through 8 *Comparator Group* Pain interference with sleep (assessed with phone report): improved for weeks 2 through 8 *Between groups* WHYMPI, Quality of sleep: no significant differences	BoNT-B administration did not show differences between groups in WHYMPI, and Quality of sleep

*Abbreviations:* BoNT-A: Botulinum Neurotoxin type A; BoNT: Botulinum Neurotoxin; BPI: Brief Pain Inventory; CG: comparator group; CPSQI: Chinese version of the Pittsburgh Sleep Quality Index; DASH: Disabilities of the Arm, Shoulder, and Hand; DN4: Douleur Neuropathique en 4 questions; EMG: electromyography; HADS: Hospital Anxiety and Depression Scale; IG: intervention group; ISCIBODS: International SCI Pain Basic Data Set; LSB: lumbar sympathetic block; NPS: Neuropathic Pain Scale; NPSI: Neuropathic Pain Symptom Inventory; NS: not significant; SCI: Spinal Cord Injury; SF-36: 36-Item Short Form Health Survey; SF-MPQ: short-form McGill Pain Questionnaire; PSQI: Pittsburgh Sleep Quality Index; WHOQOL-BREF: World Health Organization quality of life questionnaire; WHYMPI: West Haven-Yale Multidimensional Pain Inventory.

**Table 2:** BoNT administration protocols of the RCTs included in the present systematic review.

Study	Type of BoNT	Source of BoNT	Amount of BoNT	Injection Sites	Number of Injections	Rout of Injection
*Post Herpetic Neuropathy*						
Apalla et al. [36]	Onabotulinumtoxin A	Botox, Allergan	100 units	Painful area	Chessboard distribution, with a minimum distance of 1 cm between injections’ sites, 40 injections in total.	Subcutaneous
Xiao et al. [44]	NA	BoNT-A (Lanzhou Institute of Biological Products, Lanzhou, China)	Total maximum administration of 200 units	Painful area	Over the affected area, injections every 1.0–2.0 cm radius of skin.	Subcutaneous
***Peripheral nerve lesion***						
Attal et al. [26]	Onabotulinumtoxin A	Botox; Allergan	Total maximum administration of 300 units, 5 units per injection	Painful area	Up to 60 injections, at sites 1.5–2 cm apart.	Subcutaneous
***Posttraumatic/Postoperative Nerve Lesion or Post Herpetic Neuropathy***						
Ranoux et al. [41]	Onabotulinumtoxin A	Botox, Allergan	Total maximum administration of 200 units, 5 units per injection	Painful area	Up to 40 injections, at sites 1.5 cm apart in the area mapped with a pen.	Subcutaneous
***Thoracic Outlet Syndrome***						
Finlayson et al. [38]	Onabotulinumtoxin A	Botox, Allergan	75 units	Anterior and middle scalene muscles	1 injection	Intramuscular under EMG guidance
***Diabetic neuropathy***						
Ghasemi et al. [39]	Abobotulinumtoxin A	Dysport, Ipsen	100 units; each injection approximately 8–10 units	Dorsum of the foot	Grid distribution pattern covering a total of 12 (3 × 4) sites.	Subcutaneous
Salehi et al. [42]	Abobotulinumtoxin A	Dysport, Ipsen	100 units; 0.1 mL (8.33 units) injection per site	Foot surface	Grid pattern of 12 points (3 × 4).	Subcutaneous
Taheri et al. [43]	NA	NA	150 units total; Group D1 each injection 7.5 U, Group D2 each injection 3.75 units.	Sole of the foot	Twenty points at distance of 1 cm from each other (a 5 × 4 grid).	Subcutaneous
Yuan et al. [45]	Onabotulinumtoxin A	Botox, Allergan	50 units per foot; each injection 4 units	Dorsum of the foot	Grid distribution pattern covering a total of 12 (3 × 4) sites.	Subcutaneous
***Spinal Cord Injury***						
Chun et al. [37]	Onabotulinumtoxin A	Botox, Allergan	Total maximum administration of 400 units, 5 units per injection	Painful area	Up to 80 injections; the area of pain was marked using a skin marker and a plastic cut-out template for injection sites separated from each other by a 1 cm radius.	Subcutaneous
Han et al. [40]	Letibotulinumtoxin A	Meditoxin (Medytox, Seoul, Korea)	200 units	Painful area	Checkerboard pattern over the maximally affected area.	Subcutaneous
***Carpal Tunnel Syndrome***						
Breuer et al. [46]	Rimabotulinumtoxin B	Myobloc, Supernus Pharmaceuticals	2500 units divided in 3 injections	Opponens digiti minimi, flexor digiti minimi, palmaris brevis muscle	3 injections (one for each muscle)	Intramuscular under EMG guidance for opponens digiti minimi and flexor digiti minimi, and anatomically located for palmaris brevis muscle

Abbreviations: BoNT: Botulinum Neurotoxin; EMG: electromyography; cm: centimeters.

**Table 3:** Quality assessment of the studies included in the present systematic review.

Articles	Domain					Score
	Random Sequence Generation	Appropriate Randomization	Blinding of Participants or Personnel	Blinding of Outcome Assessors	Withdrawals and Dropouts	
Apalla et al. [36]	1	1	1	1	1	5
Attal et al. [26]	1	1	1	1	1	5
Breuer et al. [46]	1	0	1	1	0	3
Chun et al. [37]	1	1	1	1	1	5
Finlayson et al. [38]	1	1	1	1	1	5
Ghasemi et al. [39]	1	1	1	1	1	5
Han et al. 2016 [40]	1	1	1	1	1	5
Ranoux et al. [41]	1	1	1	1	1	5
Salehi et al. [42]	1	1	1	0	1	4
Taheri et al. [43]	1	1	1	1	0	4
Xiao et al. [44]	1	0	1	1	1	4
Yuan et al. [45]	1	0	1	1	1	4

Points were awarded as follows: study described as randomized, 1 point; appropriate randomization, 1 point; subjects blinded to intervention, 1 point; evaluator blinded to intervention, 1 point; description of withdrawals and dropouts, 1 point.

## Data Availability

The datasets generated during the current study are available from the corresponding author on reasonable request.

## Supplementary Materials

The following supporting information can be downloaded at: https://www.mdpi.com/article/10.3390/toxins14050308/s1, Table S1. Search strategy; Table S2. Records excluded.

## Abbreviations

BoNT	botulinum toxin
BoNT-A	BoNT type A
BoNT-B	BoNT type B
CENTRAL	Cochrane Central Register of Controlled Trials
HADS	Hospital Anxiety and Depression Scale
HR-QoL	health-related quality of life
NRS	Numerical Rating Scale
NS	non significant
PEDro	Physiotherapy Evidence Database
RCTs	randomized controlled trials
SNRIs	Serotonin and norepinephrine reuptake inhibitors
TENS	Transcutaneous Electrical Nerve Stimulator
TRPM8	Transient Receptor Potential Member 8
TRPV1	Transient Receptor Potential Vanilloid 1
VAS	Visual Analogue Scale
WHOQOL-BREF	World Health Organization Quality of Life questionnaire

## References

[1] Scholz J., Finnerup N.B., Attal N., Aziz Q., Baron R., Bennett M.I., Benoliel R., Cohen M., Cruccu G., Davis K.D. (2019). The IASP classification of chronic pain for ICD-11: Chronic neuropathic pain. Pain.

[2] Doth A.H., Hansson P.T., Jensen M.P., Taylor R.S. (2010). The burden of neuropathic pain: A systematic review and meta-analysis of health utilities. Pain.

[3] Bernetti A., Agostini F., de Sire A., Mangone M., Tognolo L., Di Cesare A., Ruiu P., Paolucci T., Invernizzi M., Paoloni M. (2021). Neuropathic Pain and Rehabilitation: A Systematic Review of International Guidelines. Diagnostics.

[4] Langley P.C., Van Litsenburg C., Cappelleri J.C., Carroll D. (2013). The burden associated with neuropathic pain in Western Europe. J. Med. Econ..

[5] Colloca L., Ludman T., Bouhassira D., Baron R., Dickenson A.H., Yarnitsky D., Freeman R., Truini A., Attal N., Finnerup N.B. (2017). Neuropathic pain. Nat. Rev. Dis. Primers.

[6] Van Hecke O., Austin S.K., Khan R.A., Smith B.H., Torrance N. (2014). Neuropathic pain in the general population: A systematic review of epidemiological studies. Pain.

[7] Deng Y., Luo L., Hu Y., Fang K., Liu J. (2016). Clinical practice guidelines for the management of neuropathic pain: A systematic review. BMC Anesth..

[8] Bates D., Schultheis B.C., Hanes M.C., Jolly S.M., Chakravarthy K.V., Deer T.R., Levy R.M., Hunter C.W. (2019). A Comprehensive Algorithm for Management of Neuropathic Pain. Pain Med..

[9] Baron R., Binder A., Wasner G. (2010). Neuropathic pain: Diagnosis, pathophysiological mechanisms, and treatment. Lancet Neurol..

[10] Braz J., Solorzano C., Wang X., Basbaum A.I. (2014). Transmitting pain and itch messages: A contemporary view of the spinal cord circuits that generate gate control. Neuron.

[11] Tsuda M., Koga K., Chen T., Zhuo M. (2017). Neuronal and microglial mechanisms for neuropathic pain in the spinal dorsal horn and anterior cingulate cortex. J. Neurochem..

[12] Nelson T.S., Fu W., Donahue R.R., Corder G.F., Hokfelt T., Wiley R.G., Taylor B.K. (2019). Facilitation of neuropathic pain by the NPY Y1 receptor-expressing subpopulation of excitatory interneurons in the dorsal horn. Sci. Rep..

[13] Papuc E., Rejdak K. (2013). The role of neurostimulation in the treatment of neuropathic pain. Ann. Agric. Environ. Med..

[14] Wu G., Ringkamp M., Hartke T.V., Murinson B.B., Campbell J.N., Griffin J.W., Meyer R.A. (2001). Early onset of spontaneous activity in uninjured C-fiber nociceptors after injury to neighboring nerve fibers. J. Neurosci..

[15] Caterina M.J., Julius D. (2001). The vanilloid receptor: A molecular gateway to the pain pathway. Ann. Rev. Neurosci..

[16] Serra J., Sola R., Quiles C., Casanova-Molla J., Pascual V., Bostock H., Valls-Sole J. (2009). C-nociceptors sensitized to cold in a patient with small-fiber neuropathy and cold allodynia. Pain.

[17] Wasner G., Schattschneider J., Binder A., Baron R. (2004). Topical menthol—A human model for cold pain by activation and sensitization of C nociceptors. Brain.

[18] Chaparro L.E., Wiffen P.J., Moore R.A., Gilron I. (2012). Combination pharmacotherapy for the treatment of neuropathic pain in adults. Cochrane Database Syst. Rev..

[19] Smith E.S.J. (2018). Advances in understanding nociception and neuropathic pain. J. Neurol..

[20] De Sire A., Ammendolia A., Lippi L., Farì G., Cisari C., Invernizzi M. (2021). Percutaneous Electrical Nerve Stimulation (PENS) as a Rehabilitation Approach for Reducing Mixed Chronic Pain in Patients with Musculoskeletal Disorders. Appl. Sci..

[21] De Sire A., Lippi L., Curci C., Calafiore D., Cisari C., Ammendolia A., Invernizzi M. (2021). Effectiveness of Combined Treatment Using Physical Exercise and Ultrasound-Guided Radiofrequency Ablation of Genicular Nerves in Patients with Knee Osteoarthritis. Appl. Sci..

[22] Harden N., Cohen M. (2003). Unmet Needs in the Management of Neuropathic Pain. J. Pain Symptom Manag..

[23] Intiso D., Basciani M., Santamato A., Intiso M., Di Rienzo F. (2015). Botulinum Toxin Type A for the Treatment of Neuropathic Pain in Neuro-Rehabilitation. Toxins.

[24] Fishman L.M., Anderson C., Rosner B. (2002). BOTOX and physical therapy in the treatment of piriformis syndrome. Am. J. Phys. Med. Rehabil..

[25] Baricich A., Picelli A., Carda S., Smania N., Cisari C., Santamato A., de Sire A., Invernizzi M. (2019). Electrical stimulation of antagonist muscles after botulinum toxin type A for post-stroke spastic equinus foot. A randomized single-blind pilot study. Ann. Phys. Rehabil. Med..

[26] Attal N., de Andrade D.C., Adam F., Ranoux D., Teixeira M.J., Galhardoni R., Raicher I., Uceyler N., Sommer C., Bouhassira D. (2016). Safety and efficacy of repeated injections of botulinum toxin A in peripheral neuropathic pain (BOTNEP): A randomised, double-blind, placebo-controlled trial. Lancet Neurol..

[27] Foster L., Clapp L., Erickson M., Jabbari B. (2001). Botulinum toxin A and chronic low back pain: A randomized, double-blind study. Neurology.

[28] Park H.J., Lee Y., Lee J., Park C., Moon D.E. (2006). The effects of botulinum toxin A on mechanical and cold allodynia in a rat model of neuropathic pain. Can. J. Anaesth..

[29] Finnerup N.B., Attal N., Haroutounian S., McNicol E., Baron R., Dworkin R.H., Gilron I., Haanpaa M., Hansson P., Jensen T.S. (2015). Pharmacotherapy for neuropathic pain in adults: A systematic review and meta-analysis. Lancet Neurol..

[30] Moisset X., Bouhassira D., Avez Couturier J., Alchaar H., Conradi S., Delmotte M.H., Lanteri-Minet M., Lefaucheur J.P., Mick G., Piano V. (2020). Pharmacological and non-pharmacological treatments for neuropathic pain: Systematic review and French recommendations. Rev. Neurol..

[31] Morra M.E., Elgebaly A., Elmaraezy A., Khalil A.M., Altibi A.M., Vu T.L., Mostafa M.R., Huy N.T., Hirayama K. (2016). Therapeutic efficacy and safety of Botulinum Toxin A Therapy in Trigeminal Neuralgia: A systematic review and meta-analysis of randomized controlled trials. J. Headache Pain.

[32] Do T.M., Unis G.D., Kattar N., Ananth A., McCoul E.D. (2021). Neuromodulators for Atypical Facial Pain and Neuralgias: A Systematic Review and Meta-Analysis. Laryngoscope.

[33] Li X.L., Zeng X., Zeng S., He H.P., Zeng Z., Peng L.L., Chen L.G. (2020). Botulinum toxin A treatment for post-herpetic neuralgia: A systematic review and meta-analysis. Exp. Med..

[34] Battista S., Buzzatti L., Gandolfi M., Finocchi C., Falsiroli Maistrello L., Viceconti A., Giardulli B., Testa M. (2021). The Use of Botulinum Toxin A as an Adjunctive Therapy in the Management of Chronic Musculoskeletal Pain: A Systematic Review with Meta-Analysis. Toxins.

[35] Dawson A., Dawson J., Ernberg M. (2020). The effect of botulinum toxin A on patients with persistent idiopathic dentoalveolar pain—A systematic review. J. Oral Rehabil..

[36] Apalla Z., Sotiriou E., Lallas A., Lazaridou E., Ioannides D. (2013). Botulinum toxin A in postherpetic neuralgia: A parallel, randomized, double-blind, single-dose, placebo-controlled trial. Clin. J. Pain.

[37] Chun A., Levy I., Yang A., Delgado A., Tsai C.Y., Leung E., Taylor K., Kolakowsky-Hayner S., Huang V., Escalon M. (2019). Treatment of at-level spinal cord injury pain with botulinum toxin A. Spinal Cord Ser. Cases.

[38] Finlayson H.C., O’Connor R.J., Brasher P.M.A., Travlos A. (2011). Botulinum toxin injection for management of thoracic outlet syndrome: A double-blind, randomized, controlled trial. Pain.

[39] Ghasemi M., Ansari M., Basiri K., Shaigannejad V. (2014). The effects of intradermal botulinum toxin type a injections on pain symptoms of patients with diabetic neuropathy. J. Res. Med. Sci..

[40] Han Z.A., Song D.H., Oh H.M., Chung M.E. (2016). Botulinum toxin type A for neuropathic pain in patients with spinal cord injury. Ann. Neurol..

[41] Ranoux D., Attal N., Morain F., Bouhassira D. (2008). Botulinum toxin type A induces direct analgesic effects in chronic neuropathic pain. Ann. Neurol..

[42] Salehi H., Moussaei M., Kamiab Z., Vakilian A. (2019). The effects of botulinum toxin type A injection on pain symptoms, quality of life, and sleep quality of patients with diabetic neuropathy: A randomized double-blind clinical trial. Iran. J. Neurol..

[43] Taheri M., Sedaghat M., Solhpour A., Rostami P., Lima B.S. (2020). The Effect of Intradermal Botulinum Toxin a injections on painful diabetic polyneuropathy. Diabetes Metab. Syndr. Clin. Res. Rev..

[44] Xiao L., Mackey S., Hui H., Xong D., Zhang Q., Zhang D. (2010). Subcutaneous injection of botulinum toxin a is beneficial in postherpetic neuralgia. Pain Med..

[45] Yuan R.Y., Sheu J.J., Yu J.M., Chen W.T., Tseng I.J., Chang H.H., Hu C.J. (2009). Botulinum toxin for diabetic neuropathic pain: A randomized double-blind crossover trial. Neurology.

[46] Breuer B., Sperber K., Wallenstein S., Kiprovski K., Calapa A., Snow B., Pappagallo M. (2006). Clinically significant placebo analgesic response in a pilot trial of botulinum B in patients with hand pain and carpal tunnel syndrome. Pain Med..

[47] Jadad A.R., Moore R.A., Carroll D., Jenkinson C., Reynolds D.J., Gavaghan D.J., McQuay H.J. (1996). Assessing the quality of reports of randomized clinical trials: Is blinding necessary?. Control. Clin. Trials.

[48] Sterne J.A.C., Savovic J., Page M.J., Elbers R.G., Blencowe N.S., Boutron I., Cates C.J., Cheng H.Y., Corbett M.S., Eldridge S.M. (2019). RoB 2: A revised tool for assessing risk of bias in randomised trials. BMJ.

[49] Egeo G., Fofi L., Barbanti P. (2020). Botulinum Neurotoxin for the Treatment of Neuropathic Pain. Front. Neurol..

[50] Oh H.M., Chung M.E. (2015). Botulinum Toxin for Neuropathic Pain: A Review of the Literature. Toxins.

[51] Park J., Chung M.E. (2018). Botulinum Toxin for Central Neuropathic Pain. Toxins.

[52] Park J., Park H.J. (2017). Botulinum Toxin for the Treatment of Neuropathic Pain. Toxins.

[53] Wei J., Zhu X., Yang G., Shen J., Xie P., Zuo X., Xia L., Han Q., Zhao Y. (2019). The efficacy and safety of botulinum toxin type A in treatment of trigeminal neuralgia and peripheral neuropathic pain: A meta-analysis of randomized controlled trials. Brain Behav..

[54] Lakra C., Cohen H. (2020). A clinical review of the use of Botulinum Toxin type A in managing central neuropathic pain in patients with spinal cord injury. J. Spinal Cord Med..

[55] Ahmed S., Subramaniam S., Sidhu K., Khattab S., Singh D., Babineau J., Kumbhare D.A. (2019). Effect of Local Anesthetic Versus Botulinum Toxin-A Injections for Myofascial Pain Disorders: A Systematic Review and Meta-Analysis. Clin. J. Pain.

[56] Forstenpointner J., Rice A.S.C., Finnerup N.B., Baron R. (2018). Up-date on Clinical Management of Postherpetic Neuralgia and Mechanism-Based Treatment: New Options in Therapy. J. Infect. Dis..

[57] Hary V., Schitter S., Martinez V. (2022). Efficacy and safety of botulinum A toxin for the treatment of chronic peripheral neuropathic pain: A systematic review of randomized controlled trials and meta-analysis. Eur. J. Pain.

[58] Van Boekel R.L.M., Vissers K.C.P., van der Sande R., Bronkhorst E., Lerou J.G.C., Steegers M.A.H. (2017). Moving beyond pain scores: Multidimensional pain assessment is essential for adequate pain management after surgery. PLoS ONE.

[59] Teasell R., Foley N., Pereira S., Sequeira K., Miller T. (2012). Evidence to practice: Botulinum toxin in the treatment of spasticity post stroke. Top. Stroke Rehabil..

[60] Baricich A., Picelli A., Santamato A., Carda S., de Sire A., Smania N., Cisari C., Invernizzi M. (2018). Safety Profile of High-Dose Botulinum Toxin Type A in Post-Stroke Spasticity Treatment. Clin. Drug Investig..

[61] Hernandez Herrero D., Miangolarra Page J.C. (2019). Descriptive analysis of the annual cost of treating spasticity with different types of botulinum toxin A. Neurologia.

[62] Smith B.H., Hebert H.L., Veluchamy A. (2020). Neuropathic pain in the community: Prevalence, impact, and risk factors. Pain.

[63] Wang C., Zhang Q., Wang R., Xu L. (2021). Botulinum Toxin Type A for Diabetic Peripheral Neuropathy Pain: A Systematic Review and Meta-Analysis. J. Pain Res..

[64] Fitzmaurice B.C., Rayen A.T.A. (2018). Treatments for neuropathic pain: Up-to-date evidence and recommendations. BJA Educ..

[65] Rhon D.I., Fritz J.M., Greenlee T.A., Dry K.E., Mayhew R.J., Laugesen M.C., Dragusin E., Teyhen D.S. (2021). Move to health-a holistic approach to the management of chronic low back pain: An intervention and implementation protocol developed for a pragmatic clinical trial. J. Transl. Med..

[66] Sdrulla A., Chen G. (2016). Minimally invasive procedures for neuropathic pain. Pain Manag..

[67] Szok D., Tajti J., Nyari A., Vecsei L. (2019). Therapeutic Approaches for Peripheral and Central Neuropathic Pain. Behav. Neurol..

[68] Varshney V., Osborn J., Chaturvedi R., Shah V., Chakravarthy K. (2021). Advances in the interventional management of neuropathic pain. Ann. Transl. Med..

[69] De Sire A., Moggio L., Demeco A., Fortunato F., Spano R., Aiello V., Marotta N., Ammendolia A. (2021). Efficacy of rehabilitative techniques in reducing hemiplegic shoulder pain in stroke: Systematic review and meta-analysis. Ann. Phys. Rehabil. Med..

[70] Cavalli E., Mammana S., Nicoletti F., Bramanti P., Mazzon E. (2019). The neuropathic pain: An overview of the current treatment and future therapeutic approaches. Int. J. Immunopathol. Pharm..

[71] Petrosino S., Schiano Moriello A. (2020). Palmitoylethanolamide: A Nutritional Approach to Keep Neuroinflammation within Physiological Boundaries-A Systematic Review. Int. J. Mol. Sci..

[72] Rowin J. (2019). Integrative neuromuscular medicine: Neuropathy and neuropathic pain: Consider the alternatives. Muscle Nerve.

[73] Leitzelar B.N., Koltyn K.F. (2021). Exercise and Neuropathic Pain: A General Overview of Preclinical and Clinical Research. Sports Med. Open.

[74] Andersen Hammond E., Pitz M., Shay B. (2019). Neuropathic Pain in Taxane-Induced Peripheral Neuropathy: Evidence for Exercise in Treatment. Neurorehabil. Neural. Repair..

[75] Mokhtari T., Ren Q., Li N., Wang F., Bi Y., Hu L. (2020). Transcutaneous Electrical Nerve Stimulation in Relieving Neuropathic Pain: Basic Mechanisms and Clinical Applications. Curr. Pain Headache Rep..

[76] Domoto R., Sekiguchi F., Tsubota M., Kawabata A. (2021). Macrophage as a Peripheral Pain Regulator. Cells.

[77] Matak I., Bolcskei K., Bach-Rojecky L., Helyes Z. (2019). Mechanisms of Botulinum Toxin Type A Action on Pain. Toxins.

[78] Lai J., Hunter J.C., Porreca F. (2003). The role of voltage-gated sodium channels in neuropathic pain. Curr. Opin. Neurobiol..

[79] Black J.A., Nikolajsen L., Kroner K., Jensen T.S., Waxman S.G. (2008). Multiple sodium channel isoforms and mitogen-activated protein kinases are present in painful human neuromas. Ann. Neurol..

[80] Siqueira S.R., Alves B., Malpartida H.M., Teixeira M.J., Siqueira J.T. (2009). Abnormal expression of voltage-gated sodium channels Nav1.7, Nav1.3 and Nav1.8 in trigeminal neuralgia. Neuroscience.

[81] Kim D.W., Lee S.K., Ahnn J. (2015). Botulinum Toxin as a Pain Killer: Players and Actions in Antinociception. Toxins.

[82] Liampas A., Rekatsina M., Vadalouca A., Paladini A., Varrassi G., Zis P. (2020). Non-Pharmacological Management of Painful Peripheral Neuropathies: A Systematic Review. Adv. Ther..

[83] Couto D.S., Goulart G., Luciano L.L., Cardoso E.J.R. (2021). Quality of life in neuropathic pain: A literature review. Res. Soc. Dev..

[84] Binder A., Baron R. (2016). The Pharmacological Therapy of Chronic Neuropathic Pain. Dtsch. Arztebl. Int..

[85] Higgins J.P., Thomas J., Chandler J., Cumpston M., Li T., Page M.J., Welch V.A. (2021). Cochrane Handbook for Systematic Reviews of Interventions Version 6.2.

[86] Page M.J., McKenzie J.E., Bossuyt P.M., Boutron I., Hoffmann T.C., Mulrow C.D., Shamseer L., Tetzlaff J.M., Akl E.A., Brennan S.E. (2021). The PRISMA 2020 statement: An updated guideline for reporting systematic reviews. BMJ.

[87] Huang X., Lin J., Demner-Fushman D. (2006). Evaluation of PICO as a knowledge representation for clinical questions. AMIA Annu. Symp. Proc..

