# Supplementary material for: Multidimensional Effectiveness of Botulinum Toxin in Neuropathic Pain: A Systematic Review of Randomized Clinical Trials
Source: Toxins (Basel). 2022 Apr 27;14(5):308. doi: 10.3390/toxins14050308 (PMC9145715; doi:10.3390/toxins14050308)
Supplement: Supplementary file 1 [file toxins-14-00308-s001.zip › toxins-1698083-supplementary.pdf]

toxins

MDPI

Review

Multidimensional effectiveness of Botulinum Toxin Type A and Type B in Neuropathic Pain: a Systematic Review of Randomized Clinical Trials

Lorenzo Lippi, Alessandro de Sire, Arianna Folli, Francesco d'Abrosca, Elisa Grana, Alessio Baricich, Stefano Carda and Marco Invernizzi

Table S1. Search strategy.

PubMed:<br>(Botulinum toxin) AND (neuropathic pain)
Scopus:<br>TITLE-ABS-KEY (botulinum toxin AND neuropathic pain)
Web of Science:<br>TS=(botulinum toxin AND neuropathic pain)
Cochrane Central Register of Controlled Trials (CENTRAL):<br>(Botulinum toxin):ti,ab,kw AND (Neuropathic pain):ti,ab,kw
Physiotherapy Evidence Database (PEDro):<br>botulinum toxin*neuropathic pain

Table S2. Characteristics of excluded studies assessed in full-text.

Study	***Reason for exclusion***
Allan et al. 2020 (NCT04585620)	Study protocol, ongoing trial
Anand et al. 2009	Congress abstract
Apfel et al. 2009	Not randomized controlled trial
Ashkenazi et al. 2009	Not randomized controlled trial
Attal et al. 2016	Congress abstract
Baron et al. 2016	Not randomized controlled trial
Borodic et al. 2002	Not randomized controlled trial
Bublak 2016	Language other than English
Carroll et al. 2009	No multidimensional assessment of pain
Cartagena et al. 2016	Not randomized controlled trial
Chaurand et al. 2017	Not randomized controlled trial
Chen et al. 2013	No multidimensional assessment of pain
Chien et al. 2010	Not neuropathic pain
Cho et al. 2019 (KCT0004617)	Study protocol, results not published
Chun et al. 2019	Congress abstract
Chung et al. 2015 (NCT02460107)	Study protocol, results not published
Climent J M et al. 2013	Not randomized controlled trial
Cuadrado M L et al. 2016	Not randomized controlled trial
De Andrés et al. 2010	Not neuropathic pain
De la Torre Canales et al. 2021	Not neuropathic pain
De Sousa E J S et al. 2019	Not randomized controlled trial
Dessy et al. 2014	Not randomized controlled trial
Diener et al. 2017	Not randomized controlled trial
Ding et al. 2017	Not randomized controlled trial
Donisi et al. 2020	Not randomized controlled trial
Eitner et al. 2017	Language other than English
Emad et al. 2011	Not randomized controlled trial
Ethans et al. 2013 (NCT01911377)	Study protocol, results not published
Fedotov et al. 2017	Language other than English
Finiels et al. 2016	Not randomized controlled trial
Fishman et al. 2017	No multidimensional assessment of pain
Gabriel et al. 2015	Not neuropathic pain
Gharib et al. 2020	Not randomized controlled trial
Helmy et al. 2021	Not randomized controlled trial
Hu et al. 2020	Not randomized controlled trial
Ipsen Medical Director 2018 (NCT03663101)	Study protocol, results not published
Jacoby et al. 2009	Congress abstract
Jain et al. 2018	Not randomized controlled trial
Kaisler et al. 2020	Language other than English
Kaufman et al. 2009	Not randomized controlled trial
Kim et al. 2021	Not randomized controlled trial
Kumada et al. 2012	Study involving animals
Lee et al. 2018	Not randomized controlled trial
Lessard et al. 2018	Not randomized controlled trial
Li et al. 2017	Retired for plagiarism
Marcolla et al. 2021	Not randomized controlled trial
Miller et al. 2009	Not neuropathic pain
Moericke et al. 2014	Congress abstract
Naderinabi et al. 2017	Not neuropathic pain
Nemoto 2014	Study involving animals
No author 2017	Not randomized controlled trial
Ranoux et al. 2011 (NCT01325090)	Study protocol, results not published
Restivo et al. 2017	Not randomized controlled trial
Rostami et al. 2015	Not randomized controlled trial
Rostami et al. 2016	Not randomized controlled trial
Royal Mike et al. 2003	Not randomized controlled trial
Seo et al. 2013	Not neuropathic pain
Singh et al. 2010	Not neuropathic pain
Tassorelli et al. 2006	Not neuropathic pain

Taylor et al. 2006	Not randomized controlled trial
Terlemez et al. 2019	Not randomized controlled trial
Torgovnick et al. 2009	Not randomized controlled trial
Torgovnick et al. 2010	Not randomized controlled trial
Uchida et al. 2009	Study involving animals
Wallegren et al. 2020	Not randomized controlled trial
Wang et al. 2021	Not randomized controlled trial
Wissel et al. 2016	Not neuropathic pain
Wittekindt et al. 2006	Not randomized controlled trial
Wu et al. 2012	No multidimensional assessment of pain
Yaraghi et al. 2018	Not neuropathic pain
